# Fine Mapping of the Affecting Tillering and Plant Height Gene *CHA-1* in Rice

**DOI:** 10.3390/plants12071507

**Published:** 2023-03-30

**Authors:** Tengkui Chen, Wuming Xiao, Cuihong Huang, Danhua Zhou, Yongzhu Liu, Tao Guo, Zhiqiang Chen, Hui Wang

**Affiliations:** National Engineering Research Center of Plant Space Breeding, South China Agricultural University, Guangzhou 510642, China

**Keywords:** lipase, rice, spatial mutagenesis, strigolactones

## Abstract

The plant architecture of rice is an important factor affecting yield. Strigolactones (SLs) are newly discovered carotenoid-derived plant hormones that play an important role in rice plant architecture. In this study, a high-tillering dwarf mutant, *CHA-1,* was identified by spatial mutagenesis. *CHA-1* was located in the region of 31.52–31.55 MB on chromosome 1 by map-based cloning. Compared with the wild-type THZ, the *CHA-1* mutant showed that ACCAC replaced TGGT in the coding region of the candidate gene *LOC_Os01g54810*, leading to premature termination of expression. Genetic complementation experiments proved that *LOC_Os01g54810* was *CHA-1*, which encodes a putative member of Class III lipase. Expression analysis showed that *CHA-1* was constitutively expressed in various organs of rice. Compared with those in THZ, the expression levels of the *D17* and *D10* genes were significantly downregulated in the *CHA-1 mutant*. In addition, the concentrations of ent-2′-epi-5-deoxystrigol (epi-5DS) in the root exudates of the *CHA-1* mutant was significantly reduced compared with that of THZ, and exogenous application of GR24 inhibited the tillering of the *CHA-1* mutant. These results suggest that *CHA-1* influences rice architecture by affecting SL biosynthesis.

## 1. Introduction

Rice (*Oryza sativa* L.) is one of the most important food crops in the world and plays an important role in global food production and consumption [1]. More than half of the world’s population depends on rice as a staple food [2]. Improving rice yield is still one of the main goals of rice breeding. Rice plant type is mainly determined by plant height, tillering and panicle structure, which directly affect the lodging resistance and yield potential of rice varieties [3].

The tillering development of rice is regulated by the initiation and outgrowth of axillary buds [4]. *MOC1* is the first cloned key gene that positively regulates rice tillering and encodes plant-specific GRAS family proteins, which activate axillary buds and promote tiller bud outgrowth [5]. *MOC3* encodes a protein with the highest homology to Arabidopsis WUS, which is a key factor in tiller bud initiation and regulates tiller bud outgrowth. *FON1* positively regulates the outgrowth of tillering buds [6,7]. *MOC3* binds to the promoter of *FON1* and activates its expression. As a coactivator of *MOC3*, *MOC1* enhances *FON1* expression in the presence of *MOC3* [8].

Plant hormones such as gibberellins (GAs), cytokinins (CKs), brassinosteroids (BRs), auxins, and strigolactones (SLs) are involved in the regulation of plant structure in rice. Among them, SLs are plant hormones, which have been discovered in recent years, that are produced by plant roots and transported upward to axillary buds, playing an important role in inhibiting the outgrowth of axillary buds [9,10].

The mutation in SL biosynthesis or signal led to plant dwarfing and increased tiller number [11]. *D27*, *D17*/*HTD1* and *D10* are key genes in the SL biosynthesis pathway in rice [12]. *D27* encodes an iron-containing protein that converts 9-trans-β-carotenone into 9-cis-β-carotenone [13]. *D17* encodes carotenoid cleavage dioxygenase 7 (*CCD7*), which converts 9-cis-β-carotene to 9-cis-β-apo-10′-carotenone [14]. *D10*, which encodes carotenoid cleavage dioxygenase 8 (*CCD8*), is located downstream of *CCD7* and converts 9-cis-10′-deoxy-β-daucosterol into a conserved endogenous precursor of SLs—caractone (CL) [14,15].

*D14*, *D3,* and *D53* play a major role in the perception and signal transduction of SLs [11]. *D53* encodes a protein similar to Clp ATPase I, which is an inhibitor of the SL signaling pathway [16]. *D3* encodes an F-box protein rich in leucine repeats, and *D53* is the ubiquitination substrate of *D3* [17]. *D14* encodes a protein of the α/β-hydrolase superfamily, which can hydrolyze SLs to form covalently linked intermediate molecules (CLIMs) and combine with CLIM as a receptor [18,19]. SLs promote the interaction of *D14* with *D53* and the F-box protein *D3*, which induces ubiquitination and the degradation of *D53*, thereby inhibiting plant branching [17]. Ideal plant structure 1 (*IPA1*) encodes a member of the SQUAMOSA promoter-binding protein-like (SPL) family transcription factor *SPL14* and acts as the direct downstream component targeted by *D53* to regulate the SL response and SL-induced gene expression in rice [20]. Although several genes related to the SL pathway have been cloned, the upstream genes affecting the SL pathway are still limited.

Lipase has the function of degrading triacylglycerol to glycerols and free fatty acids, which are widely distributed in animals, plants, and prokaryotes [21]. According to the amino acid structure characteristics of lipases, lipases can be divided into GxSxG (Gly-x-Ser-x-Gly) motif lipases and GDSL (Gly-Asp-Ser-Leu) motif lipases. Many GDSL lipases have been characterized in plants and participate in hormone signal transduction, cuticle formation, xylan deacetylation, and secondary metabolism [22]. Class III lipase is a member of the lipase family and plays a crucial role in many biological reactions such as lipid degradation, esterification, and transesterification [21]. Class III lipase plays an important role in plant growth and development. In rice, *EG1* encodes a class III lipase family protein, which is involved in the regulation of lipid metabolism in floral organs and affects the development of floral organs and spikelets [23].

The space environment has the characteristics of high vacuum, microgravity, a weak magnetic field, and complex radiation [24]. A complex space environment can lead to mutations in plant phenotypes, cell structures, genetic material, and proteins [24,25]. Space mutation technology is an effective way to develop new varieties of crops [26].

In this study, the high-tillering dwarf mutant *CHA-1* was obtained by space mutagenesis. Compared with THZ, the *CHA-1* mutant showed dwarfing, high tillering, small panicles, and poor pollen fertility. We used the F_2_ population for fine mapping to locate *CHA-1* encoding lipase III, which is a new mutation site obtained by spatial mutagenesis. Our results demonstrate that *CHA-1* influences rice architecture through the SL biosynthesis pathway. Identification of mutant *CHA-1* and cloning of the high-tillering dwarf gene *CHA-1* provide germplasm resources for rice.

## 2. Results

### 2.1. Phenotypic Analysis of the CHA-1 Mutant

**Figure 1:**
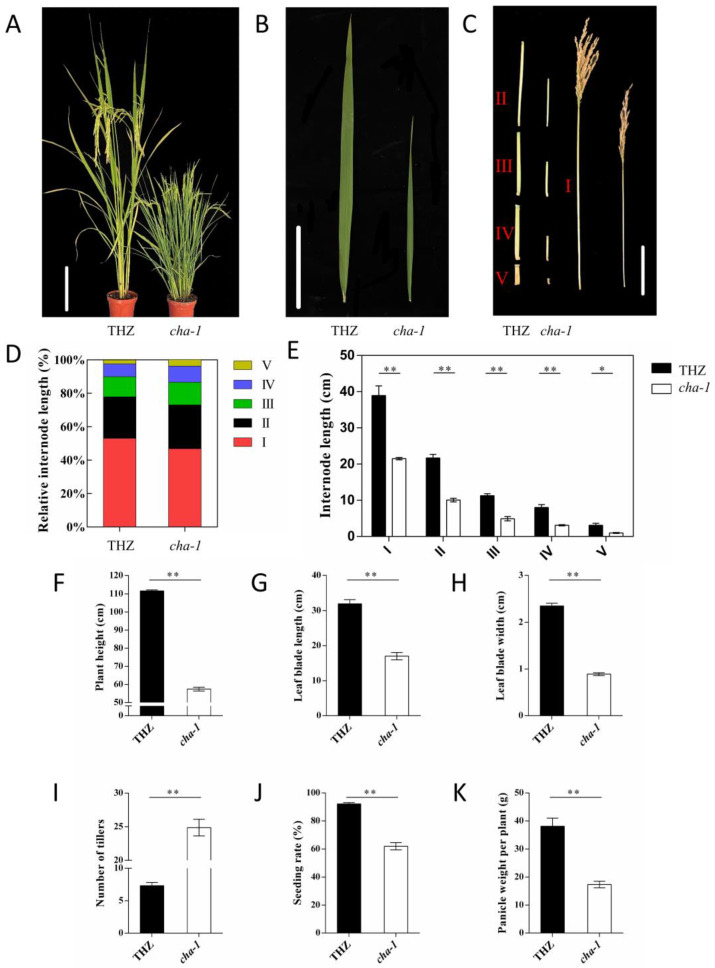
Morphological characteristics of THZ and *CHA-1*. (**A**) Morphology of THZ and *CHA-1* plants at the mature stage. Scale bar, 20 cm. (**B**) Leaf blade morphology of THZ and *CHA-1*. Scale bar, 10 cm. (**C**) Internode morphology of THZ and *CHA-1*. Scale bar, 10 cm. I, II, III, IV, and V represent internode I, internode II, internode III, internode IV, and internode V, respectively. (**D**) Comparison of the internode length ratio of THZ and *CHA-1.* (**E**) Comparison of the internode length of THZ and *CHA-1.* (**F**) Plant height. (**G**) Leaf blade length. (**H**) Leaf blade width. (**I**) Number of tillers. (**J**) Seeding rate. (**K**). Panicle weight per plant. Values are means ± SD. *, *p* < 0.05; **, *p* < 0.01; ns, no significance (Student’s *t*-test).

In previous studies, the high-tillering dwarf mutant *CHA-1* was identified from the SP2 population of *indica* rice variety Te-Hua-Zhan (THZ) induced by high-altitude ballooning, and the mutant gene *CHA-1* was found to be linked with the marker RM302 [27,28]. Compared with THZ, *CHA-1* exhibited lower plant height, increased tiller number, lower seeding rate, and shorter and thinner leaves (Figure 1A,B,F–K). The tillering dynamics of *CHA-1* and THZ showed that there was no significant difference in the early tillering stage. After seedling growth for five weeks, the tiller number of THZ increased slowly and peaked at the eighth week, while the tiller number of *CHA-1* increased rapidly after the fifth week and peaked at the eighth week (Supplementary Figure S1A–D). Finally, the tiller number of *CHA-1* was more than twice that of THZ.

The plant height of the *CHA-1* mutant was only approximately half that of THZ at the mature stage (Figure 1F). By comparing and analyzing the length of the upper five internodes of *CHA-1* and THZ, it was found that the length of each internode of *CHA-1* was shorter than that of THZ (Figure 1C,E). Compared with THZ, the shortening proportion of *CHA-1* increased gradually from the I internode to the Ⅴ internode (Figure 1D).

In addition, the seed setting rate of *CHA-1* reached only 61.95%. KI-I2 staining was used to assess the pollen viability, and the results showed that the pollen viability of *CHA-1* was only 62.9%, which was much lower than the 95.1% of THZ. We also found that the anthers of *CHA-1* were smaller and thinner than those of THZ (Supplementary Figure S1E–G). Therefore, the poor seed setting rate of mutant *CHA-1* may be caused by low pollen fertility.

### 2.2. Microscopic Observation of the CHA-1 Mutant

**Figure 2:**
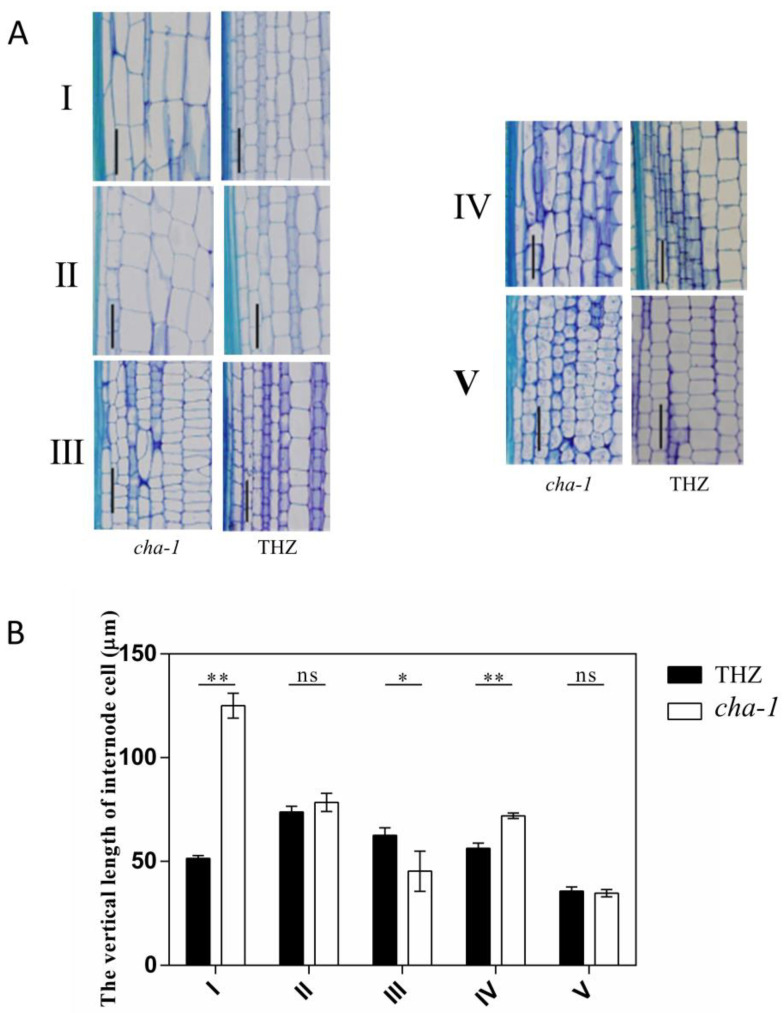
Histological comparison of internodes between THZ and *CHA-1.* (**A**) Longitudinal sections of five internodes of THZ and *CHA-1*. Scale bar, 100 μm. (**B**) Comparison of the vertical length of internode cells between THZ and *CHA-1*. Values are means ± SD. I, II, III, IV, and V represent internode I, internode II, internode III, internode IV, and internode V, respectively. *, *p* < 0.05; **, *p* < 0.01; ns, no significance (Student’s *t*-test).

To identify the factors responsible for the shortened internodes in *CHA-1*, longitudinal sections of the upper five internodes of *CHA-1* and THZ were conducted to observe and compare their internode cell length under an optical microscope. Compared with THZ, the average longitudinal length of cells in internode I, internode II, and internode IV of *CHA-1* was greater (Figure 2A,B), which indicated that the difference in internode length between THZ and mutant *CHA-1* was not due to cell size. These results suggest that the shortening of internodes was due to the reduction in the number of longitudinal cells. 

### 2.3. Fine-Mapping of CHA-1

**Figure 3:**
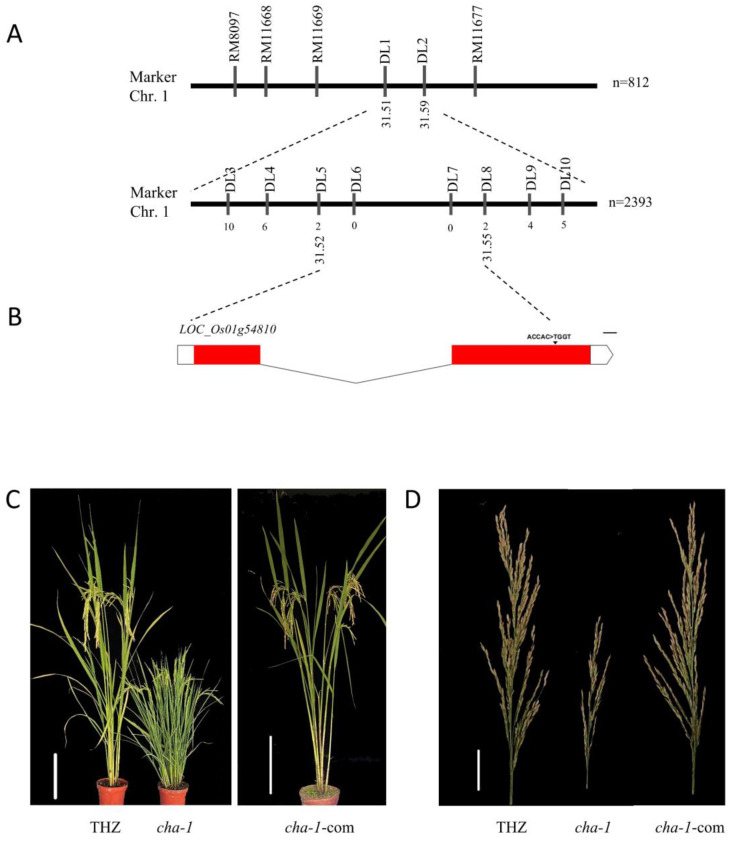
Fine mapping of *CHA-1*. (**A**) *CHA-1* was localized between markers DL10 and DL30 on chromosome 1. The name of the molecular markers are above the line, and the number below the line indicates the number of recombinants between *CHA-1* and the molecular markers shown. (**B**) Gene structure and mutation site of the candidate gene *LOC_Os01g54810*. (**C**) Morphology of THZ, *CHA-1*, and *CHA-1-com* at the mature stage. Scale bar, 20 cm. (**D**) Panicle morphology of THZ, *CHA-1*, and *CHA-1-com*. Scale bar, 10 cm. *CHA-1-com* is a complementary plant based on *CHA-1*.

The F_2_ population derived from the cross between the *CHA-1* mutant and *japonica* rice cultivar 02428 was used for fine mapping of the *CHA-1* gene. The plant height in the F_2_ population showed obvious high-dwarf separation and a continuous bimodal distribution, which indicated that there was a major gene in the *CHA-1* mutant leading to dwarfing. The separation ratio of high-dwarf plants was 3.04:1, which showed that *CHA-1* was a recessive gene (Table 1). According to the previously identified marker RM302 on the long arm of chromosome 1 [27], which was linked to *CHA-1*, we carried out further fine mapping of *CHA-1* using dwarf plants with a homozygous genotype in the F_2_ population by SSR markers and InDel markers. As a result, *CHA-1* was flanked by markers DL1 and DL2, spanning the region from 31.51 Mb to 31.59 Mb of chromosome 1 (Figure 3A). To further narrow the interval of *CHA-1*, 2393 recessive homozygous genotype plants were developed by expanding the F_2_ population to perform fine mapping. Finally, *CHA-1* was located in a region of 29.52 kb flanked by markers DL5 and DL8 (Figure 3A), which covered three candidate genes (Table 2).

### 2.4. Analysis of Candidate Genes of CHA-1

Sequencing analysis of three candidate genes within a 29.52 kb region on chromosome 1 showed that ACCAC replaced TGGT in 2382–2386 bp after the initial codon of *LOC_Os01g54810*, and the mutation site was Chr1.31524649-31524653 (-) (Figure 3B). This mutation causes code shift mutation, termination codons appear in advance, and translation terminates in advance. There was no sequence difference among other candidate genes between the *CHA-1* mutant and THZ. Therefore, *LOC_Os01g54810* was the likely candidate gene of *CHA-1*. To confirm that *LOC_Os01g54810* is *CHA-1*, we introduced a DNA fragment containing the entire genome sequence of *LOC_Os01g54810* including its upstream 2 kb sequence into the *CHA-1* background. The height, tillering, and pollen fertility of the T_1_ transgenic plants were fully restored to those of the wild type (Figure 3C,D). These results fully demonstrate that the mutation of *LOC_Os01g54810* was responsible for the phenotypic variation of mutant *CHA-1*.

### 2.5. Expression and Evolutionary Analysis of CHA-1

**Figure 4:**
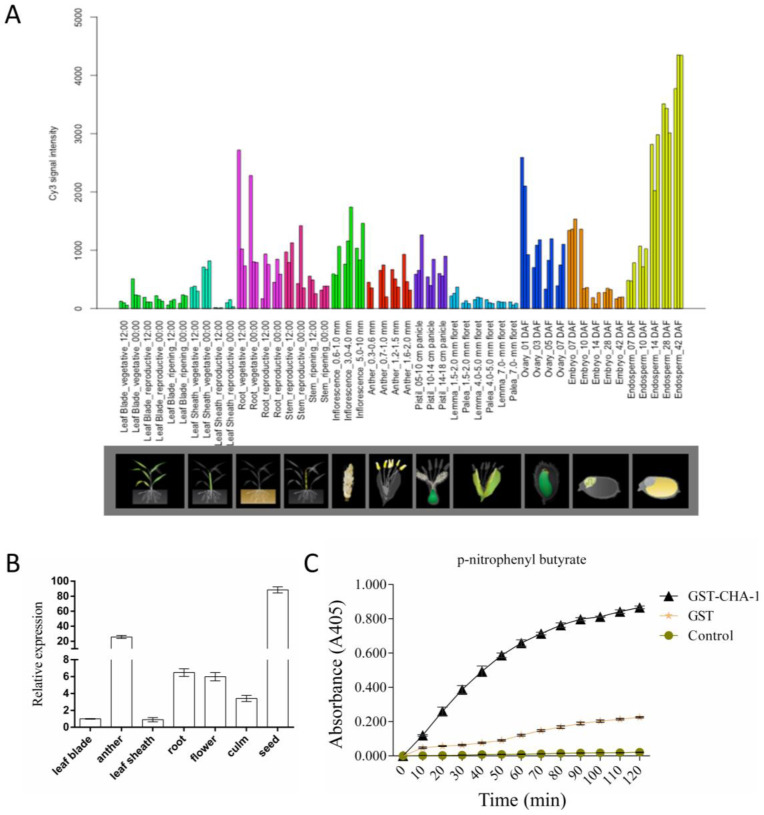
Expression analysis of *CHA-1*. (**A**) Expression patterns of the candidate gene *LOC_Os01g54810*. Gene expression profile data were obtained from the Rice Expression Profile (RiceXPro) database (http://ricexpro.dna.affrc.go.jp/, accessed on 20 February 2022). (**B**) Expression of the candidate gene *LOC_Os01g54810* in various organs of rice. (**C**) Lipase activities of *CHA-1* was incubated with p- nitrophenyl butyrate. Absorbance readings were collected every 10 min for 120 min. The substrates were incubated with GST-tagged protein and no protein as the GST and control, respectively. GST-*CHA-1* indicates that the fusion protein of the *CHA-1* and GST-tagged protein were incubated in the substrates.

*LOC_Os01g54810* encodes a putative member of Class III lipase, which hydrolyzes the ester bond of triglycerides. Using the RiceXPro database [29] (http://ricexpro.dna.affrc.go.jp/, accessed on 20 February, 2022) to analyze its expression profile, it was revealed that *LOC_Os01g54810* was expressed in multiple tissues of rice (Figure 4A). qRT–PCR was performed in several rice tissues, and the results were consistent with those in the database. *LOC_Os01g54810* was expressed in multiple tissues including the leaf lamina, leaf sheath, root, flower, culm, and seed (Figure 4B).

Phylogenetic tree analysis and amino acid sequence alignment revealed that *CHA-1* homologs were conserved in several monocots and dicots, respectively (Supplementary Figure S3). Furthermore, the *CHA-1* homology showed obvious separation between the dicot and monocot species groups in the phylogenetic tree analysis (Supplementary Figure S2), indicating that it underwent a different evolution after the differentiation of the dicot and monocot species groups.

Six *CHA-1* homologs were identified in rice. Sequence alignment showed that the N-terminal amino acid sequence of *CHA-1* was quite different from that of other homologs and that the amino acid sequence of *CHA-1* was longer (Supplementary Figure S5). This implied that *CHA-1* may have a unique biological function. *LOC_Os01g14080* and *LOC_Os01g47610* were similar to *CHA-1* in gene structure, but the CDD-Blast results showed that only *CHA-1* had the III lipase domain (Supplementary Figure S4).

In order to detect whether *CHA-1* has lipase activity, the lipase activity of the fusion protein GST-*CHA-1* was determined using p-nitrophenyl butyrate as a substrate. Compared with the control and GST alone, GST-*CHA-1* hydrolyzed the lipid substrate effectively (Figure 4C), indicating that *CHA-1* has lipase activity and is a functional lipase.

### 2.6. CHA-1 Affects the SL Biosynthesis

**Figure 5:**
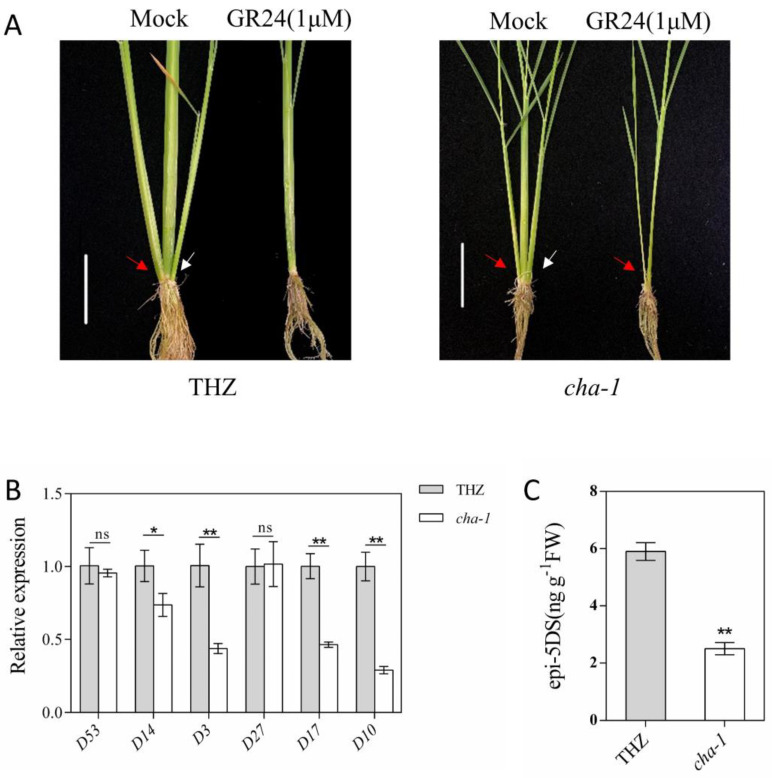
Effect of the partial loss-of-function allele of *CHA-1* on SL biosynthesis. (**A**) Length of the tiller buds of THZ and *CHA-1* treated with 1 μmol GR24 for 4 weeks. GR24 is a synthetic strigolactone. Plants were treated without GR24 as mock-treated control. (**B**) Relative expression levels of SL pathway genes between THZ and *CHA-1*. *D53*, *D14*, and *D3* were SL signaling pathway genes. *D27*, *D17*, and *D10* were SL biosynthesis pathway genes. (**C**) Comparison of epi-5DS contents in the THZ and *CHA-1* root exudates. Values are the means ± SD. *, *p* < 0.05; **, *p* < 0.01; ns, no significance (Student’s *t*-test).

In rice, mutation of the biosynthetic and signaling pathways of SLs leads to dwarfing and high-tillering phenotypes [30]. The phenotype of the *CHA-1* mutant was similar to the phenotype of mutants related to the biosynthetic and signaling pathways of SLs. Therefore, we speculated that *CHA-1* may influence the rice architecture by affecting the SL pathway. To determine whether *CHA-1* affected the SL pathway, we measured the epi-5DS content in the root exudates of THZ and *CHA-1* by HPLC-MS/MS. Compared with THZ, *CHA-1* had significantly lower amounts of epi-5DS (Figure 5C). The results of qRT–PCR analysis showed that the expression levels of the *D17*, *D10, D14*, and *D3* in the *CHA-1* mutant were significantly downregulated (Figure 5B). After 1 μmol synthetic strigolactone (GR24) treatment, the *CHA-1* and THZ tillers were inhibited; that is, the *CHA-1* phenotype with multiple tillers was rescued (Figure 5A). These results demonstrate that the mutation of *CHA-1* led to the downregulation of SL biosynthesis genes *D17* and *D10*, which resulted in the decrease in endogenous SL content.

## 3. Materials and Methods

### 3.1. Plant Materials and Phenotypic Characterization

An F_2_ population was constructed using 02428 (*Oryza sativa* L. ssp. japonica) and the *CHA-1* mutant (*Oryza sativa* L. ssp. indica) as the male and female parents, respectively. All rice populations were planted in the experimental field of the South China Agricultural University Campus Teaching & Research Base (Guangzhou, 23.16° N, 113.36° E), where there was no disease stress. There were six rows per plot, six plants per row, and a planting interval of 20 × 20 cm. Mature pollen grains were stained with 1% I2-KI solution and photographed with an Olympus CX31 light microscope (Olympus Corporation, Tokyo, Japan). Mature anthers were photographed with an Optec DV500 Digital Camera system (Optec, Chongqing, China).

### 3.2. Histological Analysis

At the mature stage of rice, five internode tissues (approximately 2–3 mm) from the stems of the main panicle were fixed with FAA (5% formaldehyde, 5% acetic acid, and 90% ethanol), dehydrated with a gradient alcohol, and then embedded into paraffin. The sections were stained with toluidine blue and observed under an Olympus IX70 light microscope (Olympus, Tokyo, Japan).

### 3.3. Fine Mapping of CHA-1

DNA samples of the F_2_ population were extracted from leaves by the cetyltrimethylammonium bromide (CTAB) method [31]. SSR marker primer sequences were obtained by using the Grame database (https://www.gramene.org, accessed on 20 February 2022) and developing new Indel marker sequences for the fine-scale localization of *CHA-1* (Table 3). Linkage analysis was performed on 3205 F_2_ plants with recessive homozygous genotypes using polymorphic markers. 

### 3.4. Phylogenetic Tree Construction and Analysis

*CHA-1* and homologous sequences were downloaded from the NCBI database. Phylogenetic trees were constructed using MEGA7.0 [32]. Sequence relationships were inferred using the maximum likelihood (ML) method. The conserved motifs were predicted using the online multiple expectation maximization for motif elicitation (MEME) program [33]. Images were generated using TBtools software [34].

### 3.5. Lipase Activity Assay

The CDS sequence of *CHA-1* was amplified by Phanta Max Super-Fidelity DNA Polymerase (Vazyme, Nanjing, Jiangsu, China) and cloned into the pGEX-4T-1 vector. The recombinant vector was transformed into *Escherichia coli* strain BL21 (DE3) and the expression of the recombinant protein was induced by isopropylthio-β-galactoside. Purification of *CHA-1* and the lipase assay were performed as described previously [35]. The recombinant protein was incubated with 1 mM p-nitrophenyl butyrate substrate in enzyme reaction buffer (0.5 M HEPES, pH 6.5) at 30 °C for 1 h. The absorbance was measured every 10 min at 405 nm for 120 min.

### 3.6. RNA Extraction and Quantitative Real-Time PCR (qPCR)

Total RNA was extracted from various organs using a TRIzol Kit (Invitrogen) following the manufacturer’s method. cDNA was reverse transcribed using 5× HiScript III qRT SuperMix (Vazyme). qPCR experiments were performed using AceQ qPCR SYBR Green Master Mix (Vazyme) and the ABI Step One Plus Real-Time PCR System (Applied Biosystems, Foster City, CA, USA). The relative expression was estimated by the 2^−ΔΔCT^ method [36].

### 3.7. Vector Construction and Transformation

The full-length DNA and upstream promoter (approximately 2 kb from the start codon) of *CHA-1* were cloned into the pCAMBIA1300 vector, which was introduced into *Agrobacterium tumefaciens* strain EHA105 and then transformed into rice callus, as previously reported [37].

### 3.8. GR24 Treatment

SL analysis in the rice root exudates was quantified by Webiolotech (Nanjing, China) as previously reported [16] with minor modifications. One-week-old seedlings were grown on hydroponic culture medium without phosphate for 14 days. The hydroponic culture medium was collected, and the fresh root weight was recorded. A total of 50 mL hydroponic culture medium was loaded into a pre-balanced Oasis HLB 3cc cartridge (Waters, Milford, MA, USA) and the column was washed with deionized water. Fractions containing SLs were eluted with acetone, collected and dried under nitrogen, then reconstituted in acetonitrile and subjected to HPLC-tandem mass spectrometry (HPLC-MS/MS) analysis. SLs analysis was performed on a quadruple linear ion trap hybrid MS (QTRAP 6500, AB SCIEX) equipped with an electrospray ionization source coupled with a HPLC (Aglient1290, Aglient, Santa Clara, CA, USA).

Hydroponic culture medium preparation and GR24 (Coolaber, Beijing, China) treatment were performed as previously described [9]. The 1-week-old seedlings were grown in a climatic cabinet under 16 h light at 28 °C and 8 h dark at 25 °C for another 28 days, and the hydroponic culture medium with or without 1 μmol GR24 was renewed every week.

## 4. Discussion

Compared with other physical mutations, space mutation has the characteristics of non-replication. Each space mutation is subject to different spatial radiation, and the obtained mutants showed various types with different frequencies [25]. Space mutation breeding has a unique advantage and role in creating excellent new germplasms, inducing new gene resource mutations and cultivating new varieties of crops, which is an effective method for crop genetic improvement [24,26,38,39].

In a previous study, the *this1* mutant showed a phenotype with dwarfing, high-tillering, and poor pollen fertility, which was similar to that of the *CHA-1* mutant, and *THIS1* was found to be *LOC_Os01g54810* [40]. In this study, the *CHA-1* mutation site was not consistent with the *this1* mutation site. *LOC_Os01g54810* in the *this1* mutant had a 356-bp deletion covering the intron and the second exon, while *LOC_Os01g54810* in the *CHA-1* mutant had a mutation of ACCAC replaced TGGT on the second exon. There was no genotype similar to the *CHA-1* mutant in the locus of *LOC_Os01g54810* in approximately 3000 natural varieties of rice using the Rice Functional Genomics and Breeding (RFGB) database [41] (http://www.rmbreeding.cn/, accessed on 20 February 2022). Therefore, *CHA-1* was identified as a new allele of *THI1S1* obtained by space mutation in this study.

At present, several genes related to the SL pathway have been identified, mainly including the SL signaling genes *D53*, *D14,* and *D3* and SL biosynthesis genes *D27*, *D17,* and *D10* [11]. In the mutant of SL signaling genes, SL biosynthesis genes are upregulated by negative feedback regulation [17,42]. In *d53*, *d14*, and *d27* mutants, the expression of the *D10* gene was significantly upregulated by feedback regulation [42]. In contrast, the expression of *D10* was significantly downregulated in the *d17* mutant [30]. The *CHA-1* mutant showed the phenotype of dwarfing and high tillering (Figure 1A,B), which was similar to the phenotype of the mutant associated with the SL pathway. Histological analysis of the *d53* mutant showed that the shortening and thinning of the stem of the *d53* mutant were mainly caused by the decrease in cell number [17]. Histological analysis of the *CHA-1* mutant and THZ also proved that the difference in stem node length was associated with the number of cells (Figure 2A,B). The expression of SL biosynthesis genes was downregulated in the *CHA-1* mutant (Figure 5B). The *CHA-1* mutant had significantly lower concentrations of epi-5DS in the root exudates, and the tillering of the *CHA-1* mutant was inhibited after GR24 treatment (Figure 5A,C). Therefore, *CHA-1* influences rice architecture by affecting SL biosynthesis.

Lipase regulates plant disease resistance and floral organ development by affecting the auxin pathway [35,43,44]. Auxin positively regulates the expression of SL biosynthesis genes *D17* and *D10* in Arabidopsis and rice [45]. The mutation of *CHA-1* led to the downregulation of *D17* and *D10* expression without affecting *D27* expression (Figure 5B). The *CHA-1* mutant has some mutant phenotypes that SL pathway mutants do not have. The expression pattern of *CHA-1* regulated SL biosynthesis genes is similar to that of auxin regulated SL biosynthesis genes. Therefore, we speculated that *CHA-1* indirectly regulates SL biosynthesis by affecting the auxin pathway, and its molecular mechanism remains to be further studied.

Several lipase genes have been identified to affect pollen fertility in rice [35,43,46]. The anthers of the *rms2* mutant with abnormal pollen atrophy resulted in complete male sterility. Cytological and genetic analysis showed that RMS2 had lipase activity and was necessary for anther development and pollen fertility [35]. *OsGELP34* encodes a putative GDSL lipase/esterase. The *osgelp34* mutant exhibits an abnormal outer wall, leading to pollen sterility [46]. *EG1* encodes a class III lipase family protein involved in the regulation of lipid metabolism in floral organs [23]. *CHA-1* encodes a III lipase protein and has lipase activity (Figure 4C). The *CHA-1* mutant showed anther atrophy, poor pollen fertility, and a decreased seed setting rate. Accordingly, *CHA-1,* as a lipase, affects anther development and pollen fertility.

## 5. Conclusions

The *CHA-1* mutant showed dwarfing, multi-tillering, and poor pollen fertility. Based on map-based cloning and candidate gene sequencing, *LOC_Os01g54810* was proven to be *CHA-1*, which regulated rice architecture and pollen fertility. *CHA-1* regulates the rice architecture by affecting strigolactone biosynthesis. The study on *CHA-1* will contribute to further research of the strigolactone pathway in rice.

## Figures and Tables

**Table 1:** Segregate pattern of the F_2_ population from the cross of *CHA-1* and 02428.

Types	Number of Plants	Ratio	χ^2^(3:1) χ^2^_0.05_ = 3.84
High plant height	2471	3.04:1	0.12
Low plant height	812		

**Table 2:** Annotations of candidate genes in the region covering *CHA-1* based on the MSU7 database.

Gene ID	Annotation
LOC_Os01g54810	Class III lipase
LOC_Os01g54850	Cyclin-like F-box domain containing protein
LOC_Os01g54860	Enoyl-CoA hydratase/isomerase

**Table 3:** Primers used for the fine mapping of *CHA-1* and quantitative real-time PCR.

Marker	Primer Sequences (5′–3′)
RM8097-F	GCTGTCACTGACCGAGCGTAGG
RM8097-R	TCGAGAGATCCAATCCAGTTTGC
RM11668-F	AGTGTCTCTGGAGTTGGGAGTGG
RM11668-R	CTGTTCTTCCAGATGGGCTTCC
RM11669-F	AAACCGTTCCAGGGAGACTGACC
RM11669-R	TCGTCTGATCCATCCATCCATCC
DL1-F	AATGCGTGGGGTTTCATCTA
DL1-R	TAGAGCATGGATAGACGGGG
DL3-F	AGCTATGTGGTTAGGTCC
DL3-R	TAGATGAGGAAGCCTAGT
DL4-F	AGTGGCTAGTCACTTACA
DL4-R	GGAAGCCTAGTATGAAGC
DL5-F	ATTCCGGTGGCGTTTTCA
DL5-R	CCACCAAAATTGTAGGGAGT
DL6-F	CTCATTGTTGCCTATGAG
DL6-R	GCACGTACGTAGTGAGAT
DL7-F	ATCAGAAGCTCCTGACTCTT
DL7-R	GCCGGAGAGGTAGTCGT
DL8-F	TCTGAACTGAATGGTTCG
DL8-R	TACAGTGGAGTCCTGCTA
DL9-F	AGAGCACCCAAGAGTTAATC
DL9-R	TGGCTGATATTGGGTATG
DL10-F	GCCTTAGAGGAGGATCTTCT
DL10-R	TTCCTTGCATCTCACGTAGG
DL2-F	AAAAGCCCACTTTGCATGAG
DL2-R	AGGTGTAACGAGAAAGCGGA
RM11677-F	GTCTTGGAGCTGAGCACCTTGG
RM11677-R	GGCCCTCCGTGTAATCCTATTCC
qPCR-D53-F	CCAAGCAGTTTGAAGCGAC
qPCR-D53-R	CCGCAAGTTTATCAAAGTCAA
qPCR-D10-F	CGTGGCGATATCGATGGT
qPCR-D10-R	CGACCTCCTCGAACGTCTT
qPCR-D14-F	CGCCTTCGTCGGCCACTC
qPCR-D14-R	TCGAACCCGCCGTGGTAGTC
qPCR-D17-F	CTGTTCTTAGCGGGGTGTTC
qPCR-D17-R	GGCGTCGAACTCGTAGAAAG
qPCR-D3-F	TTAAGGTGGAGGGTGATTGC
qPCR-D3-R	AAGATCCATCTGCCCTGTTG
qPCR-D27-F	TCTGGGCTAAAGAATGAAAAG
qPCR-D27-R	AGAGCTTGGGTCACAATCTCG
Com-*CHA-1*-F	GAGCTCGGTACCCGGGGATCCTCTTGTAATTTTTGGGTATAAATAGACCC
Com-*CHA-1*-R	ACGACGGCCAGTGCCAAGCTTTGTAAATTTTTGTGGTCCAAAGTGA
GST-*CHA-1*-F	CCGCGTGGATCCCCGGAATTCATGGCGATCGACCTGGCG
GST-*CHA-1*-R	GTCACGATGCGGCCGCTCGAGCTACTTTGCAGGGGGTGACG

## Data Availability

No new data were created or analyzed in this study. Data sharing is not applicable to this article.

## Supplementary Materials

The following supporting information can be downloaded at: https://www.mdpi.com/article/10.3390/plants12071507/s1, Supplementary Figure S1: Morphology comparison between THZ and *CHA-1* in the tiller and pollen fertility; Supplementary Figure S2: Evolutionary relationships, conserved protein motifs, and gene structure in the *CHA-1* homologs from various species; Supplementary Figure S3: The *CHA-1* homologous protein amino acid sequence alignment in various species; Supplementary Figure S4: Evolutionary relationships, conserved protein motifs, and gene structure in the *CHA-1* homologs from rice; Supplementary Figure S5: The *CHA-1* homologous protein amino acid sequence alignment in rice.
