# Supplementary material for: Fine Mapping of the Affecting Tillering and Plant Height Gene CHA-1 in Rice
Source: Plants (Basel). 2023 Mar 30;12(7):1507. doi: 10.3390/plants12071507 (PMC10096525; doi:10.3390/plants12071507)
Supplement: Supplementary file 1 [file plants-12-01507-s001.zip › plants-2256115-supplementary.pdf]

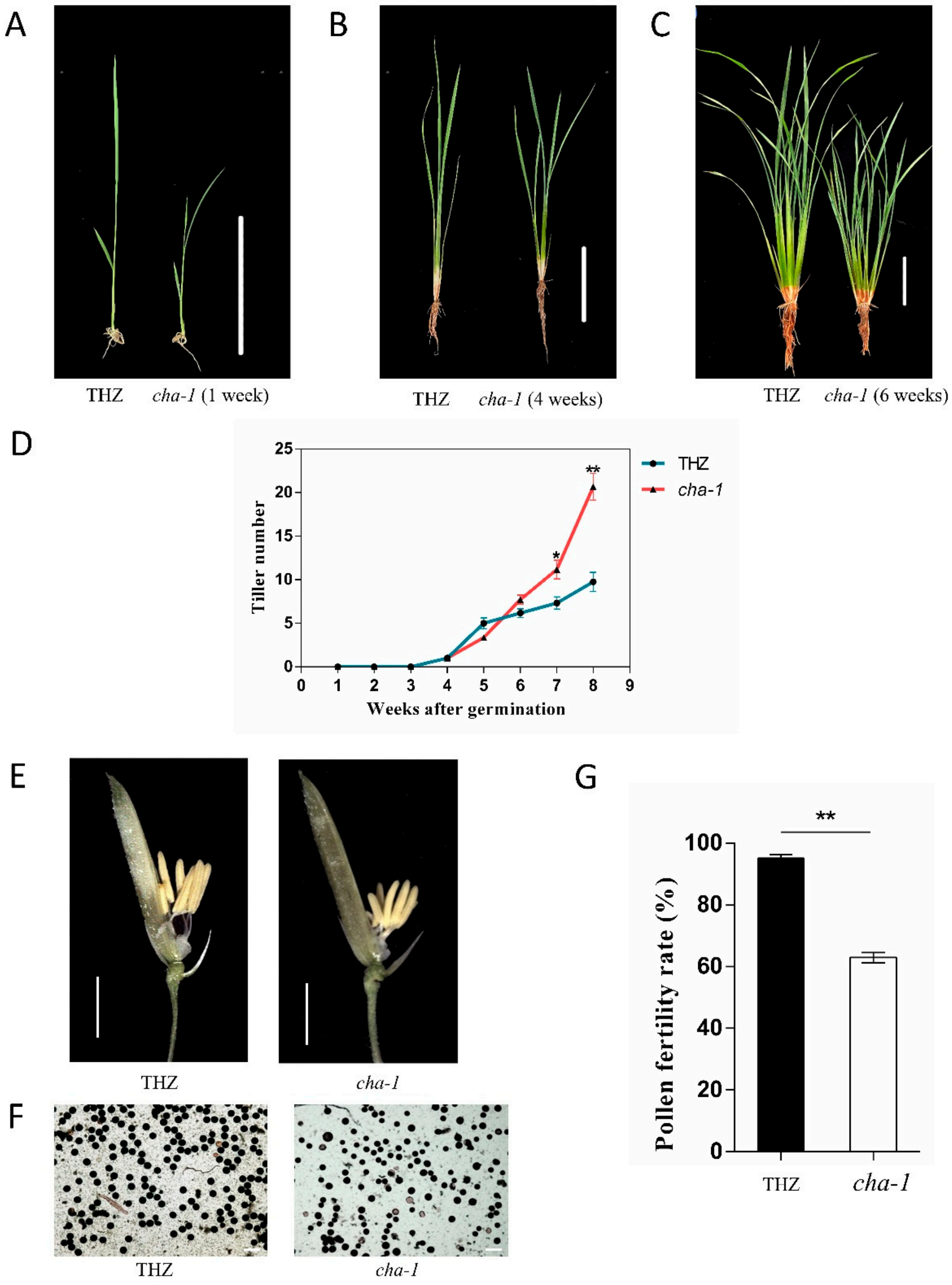

Supplementary Figure S1. Morphology comparison between THZ and *cha-1* in the tiller and pollen fertility. Morphology of the THZ and *cha-1* plants at seeding. (**A**) One week. (**B**) Four weeks. (**C**) Six weeks. (**D**) Comparison of the tillering dynamics between THZ and *cha-1*. (**E**) Anther morphology of THZ and *cha-1*. Scale bar, 2 mm. (**F**) I_2-KI assay of the mature pollen grains. Scale bar, 0.1 mm. (**G**) Comparison of the pollen fertility rates between THZ and *cha-1*.

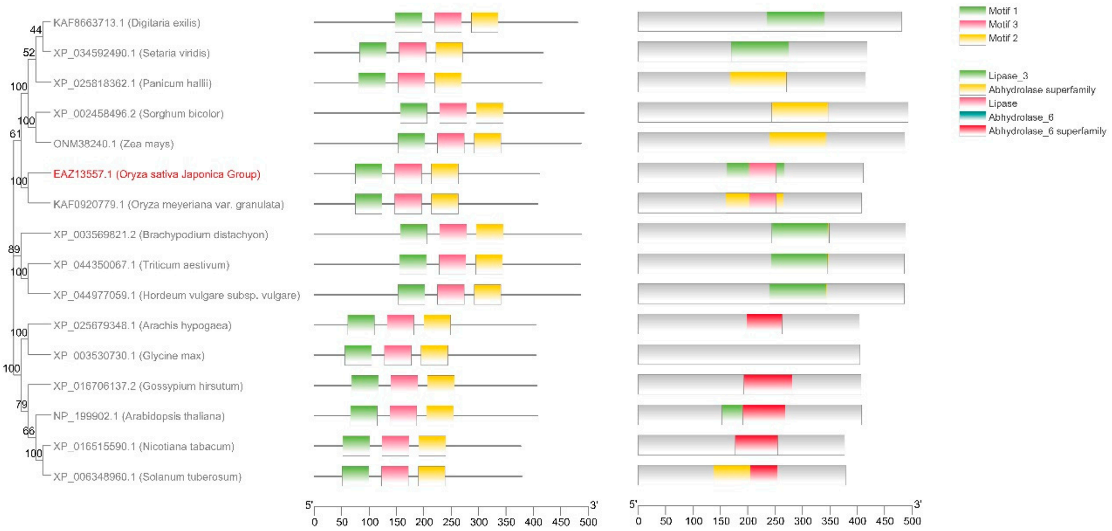

Supplementary Figure S2. Evolutionary relationships, conserved protein motifs, and gene structure in the CHA-1 homologs from various species. The phylogenetic tree was constructed using the neighbor-joining (NJ) method and 1000 bootstrap replicates. The motifs are displayed with different colored boxes in the proteins. Protein domains were predicted by CD-search.

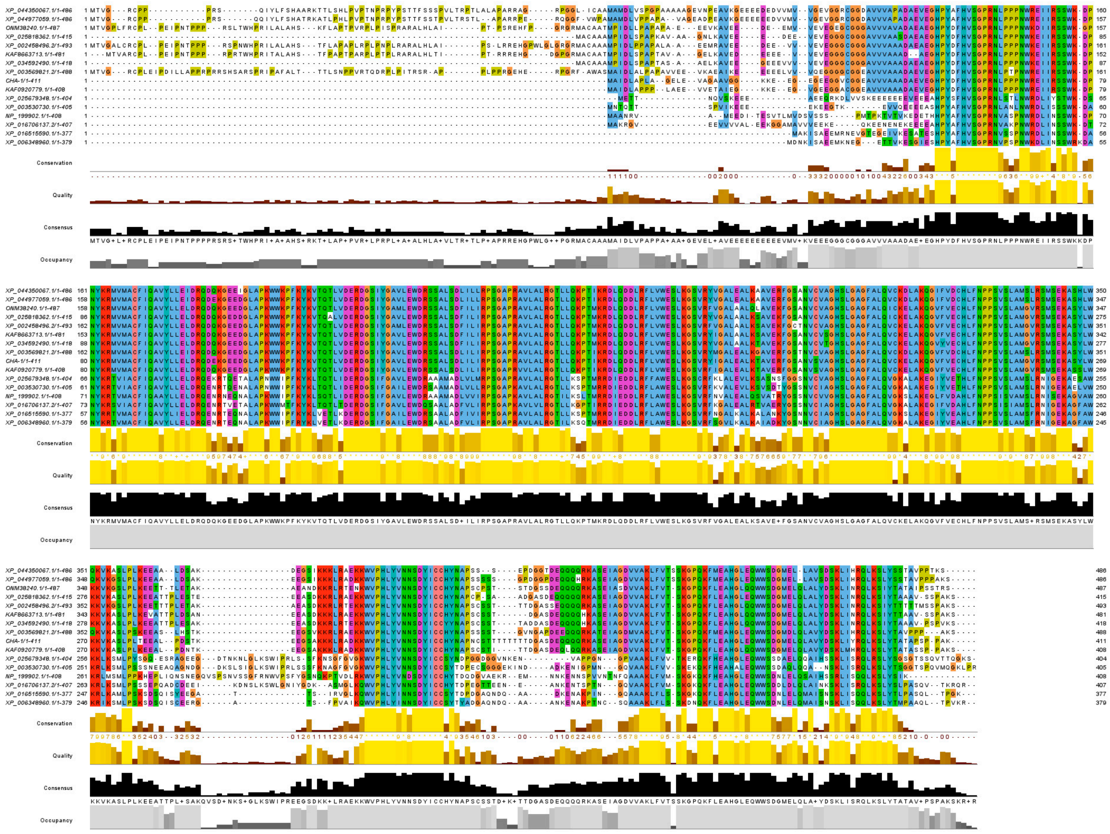

Supplementary Figure S3. The CHA-1 homologous protein amino acid sequence alignment in various species.

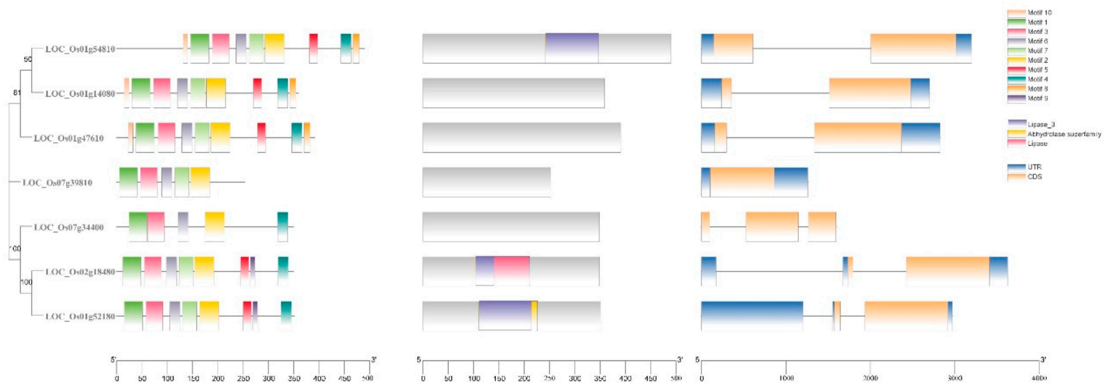

Supplementary Figure S4. Evolutionary relationships, conserved protein motifs, and gene structure in the CHA-1 homologs from rice. The phylogenetic tree was constructed using the neighbor-joining (NJ) method and 1000 bootstrap replicates. The motifs are displayed with different colored boxes in the proteins. Protein domains were predicted by CD-search. Gene structure is represented by different color boxes, with blue boxes representing the UTR and orange boxes representing the CDS region.

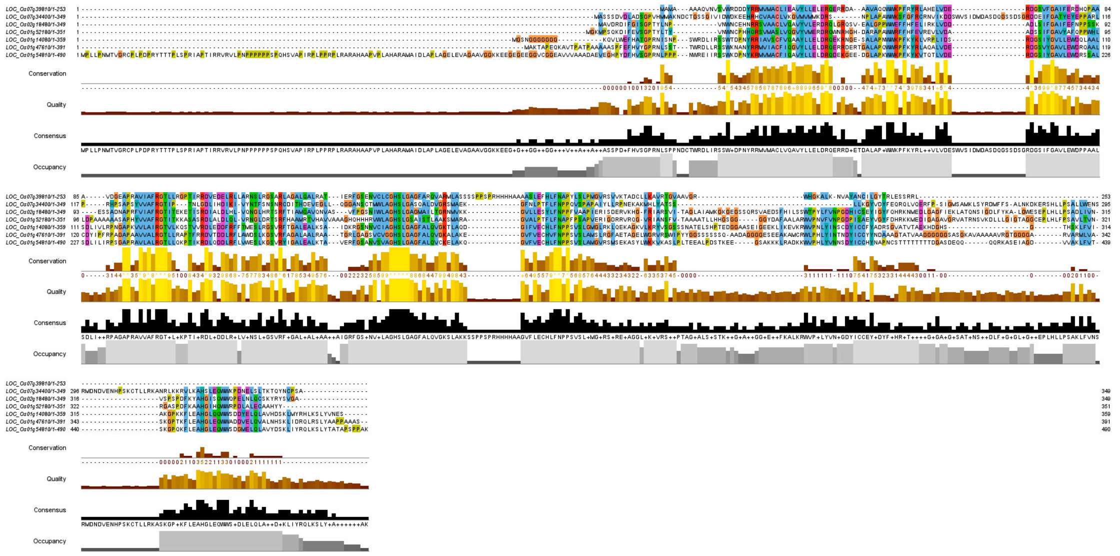

Supplementary Figure S5. The CHA-1 homologous protein amino acid sequence alignment in rice.
